# The upcoming SOFA 2.0 score: a roadmap for future developments in critical care?

**DOI:** 10.62675/2965-2774.20250067

**Published:** 2025-05-15

**Authors:** Bruno Adler Maccagnan Pinheiro Besen, Andre C Kalil, Elisa Estenssoro, Pedro Póvoa

**Affiliations:** 1 Instituto D’Or de Pesquisa e Ensino São Paulo SP Brazil Instituto D’Or de Pesquisa e Ensino - São Paulo (SP), Brazil.; 2 Postgraduate Program in Medical Sciences Faculdade de Medicina Universidade de São Paulo São Paulo SP Brazil Postgraduate Program in Medical Sciences, Faculdade de Medicina, Universidade de São Paulo - São Paulo (SP), Brazil.; 3 Division of Infectious Diseases University of Nebraska Medical Center Omaha Nebraska United States Division of Infectious Diseases, University of Nebraska Medical Center - Omaha, Nebraska, United States.; 4 Servicio de Terapia Intensiva Hospital Interzonal de Agudos San Martin de La Plata Buenos Aires Argentina Servicio de Terapia Intensiva, Hospital Interzonal de Agudos San Martin de La Plata - Buenos Aires, Argentina.; 5 Dirección de Investigación Ministerio de Salud Buenos Aires Argentina Dirección de Investigación; Ministerio de Salud - Provincia de Buenos Aires, Argentina.; 6 NOVA Medical School NOVA University of Lisboa Lisboa Portugal NOVA Medical School, NOVA University of Lisboa - Lisboa, Portugal.; 7 Research Unit of Clinical Epidemiology, Department of Clinical Research Odense University Hospital Odense Denmark Research Unit of Clinical Epidemiology, Department of Clinical Research, Odense University Hospital - Odense, Denmark.; 8 Department of Intensive Care Hospital São Francisco Xavier Lisboa Portugal Department of Intensive Care, Hospital São Francisco Xavier - Lisboa, Portugal.

## Why do we need organ dysfunction scores?

Intensive care medicine is characterized by providing organ support to dysfunctional or failing organs during acute or acute-on-chronic illness. Organ dysfunction may present in isolation or combination and occur at different times during critical illness in each patient. Although intensivists are trained to recognize and respond promptly to organ dysfunction to avoid further deterioration and, when this occurs, to provide multiple organ support, the evaluation of organ dysfunctions might differ between intensivists. Therefore, organ dysfunction scores that standardize how organ dysfunction is measured are especially warranted. They may both enhance and standardize communication by intensivists in their day-to-day interactions and aid in research purposes.

In 1996, Vincent et al. introduced the Sequential Organ Failure Assessment (SOFA) score based on a consensus definition.^(1)^The SOFA score allowed the description of organ dysfunction dynamics in patients with multiple organ failure and its prognostic association with clinical outcomes. A few are worth remembering: (i) non-recovery within a few days of organ failure onset was associated with similar outcomes to organ failure worsening;^(2)^ (ii) the maximum intensity of organ dysfunction in each domain during intensive care unit (ICU) stay (maximum total SOFA) was perhaps the strongest predictor of mortality;^(3)^ (iii) the timing of organ dysfunction was variable during acute illness, with neurological dysfunction presenting earlier, while liver and coagulation abnormalities have lagged presentations;^(3,4)^ and (iv) the prognostic role of non-neurological organ dysfunction in neurocritical care patients.^(5)^

Significantly, the SOFA score was not developed to be a prognostic score. Neither could it be, as we know that the clinical outcome of critically ill patients is only partially explained by the intensity and broadness of organ dysfunctions, with additional contribution from both patient antecedent characteristics and contextual variables reflecting ICU organizational characteristics.^(6,7)^ The SOFA score describes organ dysfunction dynamics through time, domains, and intensity.

## Why should the SOFA score be updated?

The SOFA score, initially published in 1996,^(1)^ has been successfully incorporated into critical care medicine research.^(8,9)^ It allowed descriptive and prognostic research and has been used to determine surrogate outcomes in critical care trials.^(10)^ It has also become part of the latest sepsis definitions.^(8)^ Reasons for SOFA score success perhaps involve its underlying principles: (i) inclusion of low-cost, simple, routinely collected data aiming at a low number of variables; (ii) ability to be serially measured, as organ dysfunction is dynamic throughout critical illness; and (iii) stratification of organ dysfunction in clear-cut, objectively defined, discrete categories that reflect increased organ dysfunction burden and severity. Nevertheless, although its simplicity has certainly passed the test of time and facilitated its adoption even in low-resource settings, new additions to critical care daily practice have brought the need to update the score.^(11-13)^

A few of these advancements include: (1) new technologies, either more invasive – such as venous-venous (VV-) and veno-arterial (VA-) extracorporeal membrane oxygenation (ECMO) – or less invasive – as high flow nasal oxygen (HFNO) and noninvasive ventilation (NIV) –; (2) new drugs, such as vasopressin and angiotensin II; (3) the capacity to better assess neurological dysfunction with light sedation approaches; (4) less invasive approaches to the assessment of critically ill patients, with oxygen saturation/fraction of inspired oxygen (SpO_2_/FiO_2_) ratios instead of partial pressure of oxygen/fraction of inspired oxygen (PaO_2_/FiO_2_) ratios as an example;^(14)^(5) the (virtual) abandonment of some treatment strategies (such as dopamine); and (6) identification of other possible domains to be included in the score, such as immunological dysfunction.^(15,16)^ Development and validation of an updated SOFA score is therefore the consequence of advances in critical care medicine over the last decades.

## The SOFA score is now 2.0: what are its merits?

**Figure 1:**
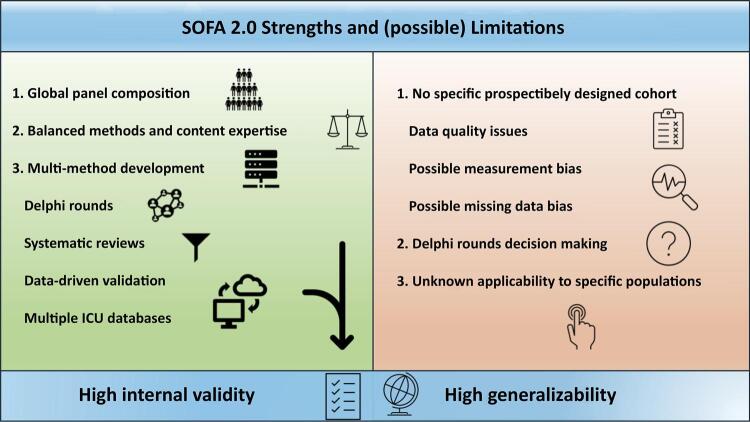
SOFA 2.0 score strengths and (possible) limitations.*

Figure 1 presents a few of the new development’s strengths and limitations. SOFA 2.0 was developed following state-of-the-art methodology, which will allow internal validity and substantial generalizability. First, the authors designed a panel composition that is more representative of the global critical care community, multinational, with representatives from all continents and from different country-level incomes. The panel also balanced content and methodological expertise, a significant strength to avoid expert opinion-based round table discussions. Second, the authors did not abandon the underlying theoretical principles to update the score. Not only has the score been based on the content expertise, which has driven Delphi rounds, but the working groups have systematically reviewed published evidence to identify possible targets to characterize the severity of organ dysfunction across each domain. Third, after considering underlying theory and published evidence, the score used a modern data-driven validation that leverages currently available real-world ICU databases. Therefore, the association of organ dysfunction categories with outcomes is based not only on theory or consensus but also on predictive validity. Finally, SOFA 2.0 is likely to be generalizable to many critical care scenarios, given the diversity in the databases to validate the cut-off points of the score.

ICU - intensive care unit.

* These strengths and limitations were considered based on past European Society of Intensive Care Medicine (ESICM) LIVES 2024 and *Congresso Brasileiro de Medicina Intensiva* (CBMI) 2024 presentations by the principal authors of the revised version.

## Are there any limitations?

SOFA 2.0 will undoubtedly come with some limitations. First, it was not based on prospective cohort studies, which may reduce data quality and completeness.^(17)^ Although large databases designed to capture clinically relevant data might partly overcome this limitation, large databases cannot necessarily prevent some sorts of bias, such as measurement bias.^(13)^ Other limitations stem from decisions made during Delphi rounds – a reflection of the choice of panelists of the working group –,^(18)^ that might be subject to bias and criticisms. Another limitation is that a single score derivation and validation may hamper generalizability to frequent and specific scenarios and subgroups, such as critical illness in pregnancy, or to underrepresented conditions from low-income countries, such as malaria and other high-burden infectious diseases of interest.

## Further research agenda

The expected publication of SOFA 2.0 in 2025 will likely come with a substantial new research agenda. Should we start using SOFA 2.0 to define sepsis instead of SOFA? What will the predictive validity of SOFA 2.0 be in defining sepsis? Will the predictive validity of SOFA 2.0 to operationally define sepsis still be based on a two-point variation of baseline SOFA? Is SOFA 2.0 applicable to specific subgroups of patients, and how are subgroups different? If new domains were not included, was there any issue in the score development approach that may be prevented in the future?

## Conclusions

The authors of SOFA 2.0 should be commended for their concerted efforts to update the SOFA score. It will hopefully be an example of a balanced panel of content experts and methodologists experienced in critical care research. Upon its publication, we expect further external prospective validation, which will be necessary to determine its accuracy and generalizability.
